# Supplementary material for: Phylodynamic assessment of intervention strategies for the West African Ebola virus outbreak
Source: Nat Commun. 2018 Jun 8;9:2222. doi: 10.1038/s41467-018-03763-2 (PMC5993714; doi:10.1038/s41467-018-03763-2)
Supplement: Supplementary file 1 — Supplementary Information [file 41467_2018_3763_MOESM1_ESM.pdf]

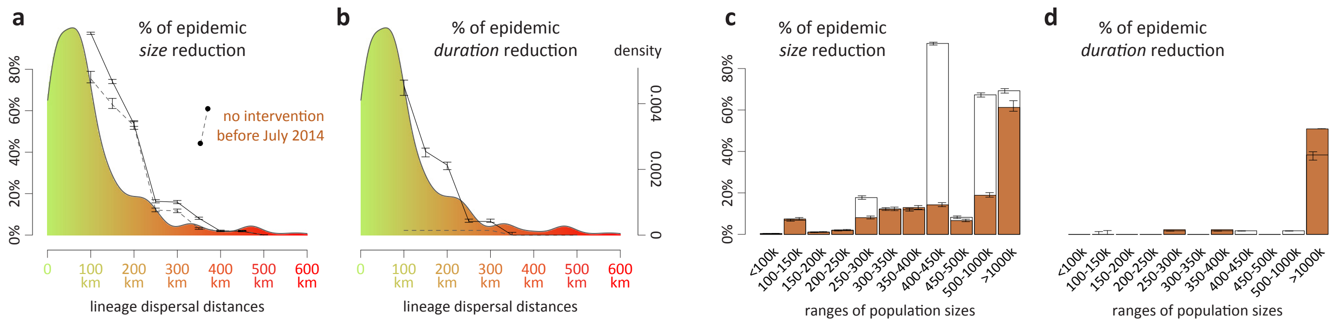


**Supplementary Figure 1. Reductions in epidemic size and duration (equivalent to Figures 1a-d) with 95% credible intervals based on 1,000 posteriori trees.** (**a**) Impact of halting transmission following long-distance dispersal events on *epidemic size*. The plot depicts the epidemic size reduction when removing transmission following dispersal events over various distances. In addition, we also report corresponding epidemic size reductions when transmission is prevented only after June 2014 (dashed line). These epidemic size reduction curves are superimposed on the distribution of lineage dispersal distances summarised based on the posterior Markov jump history. (**b**) Impact of removing transmission following long-distance dispersal events on *epidemic duration*. This plot corresponds to Supplementary Figure 1a, but focuses on the impact on epidemic duration. (**c**) Impact of preventing dispersal events to specific administrative areas on *epidemic size*. The plot reports the epidemic size reduction when removing transmission following dispersal events to administrative areas belonging to a specific population sizes range. As for Supplementary Figure 1a, we also report corresponding epidemic size reductions when transmission is prevented only after June 2014 (brown histogram). (**d**) Impact of removing transmission following dispersal events to specific administrative areas on the *epidemic duration*.

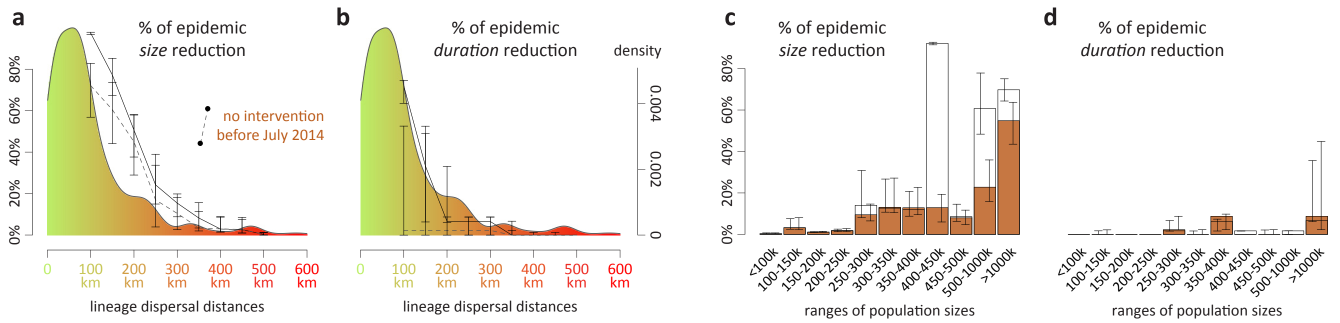


**Supplementary Figure 2. Reductions in epidemic size and duration (equivalent to Figures 1a-d) without conditioning on a single MCC tree.** Epidemic size/duration reductions reported in Figures 1a-d (and in Supplementary Figures 1a-d) are based on posterior trees pruned according to Markov jumps recorded in the MCC (maximum clade credibility) tree. To assess the sensitivity of these estimates to conditioning on a single MCC tree, we also pruned posterior trees according to their specific Markov jump histories. For computational reasons, we restricted this pruning to 360 (instead of 1,000) trees sampled from the posterior distribution of the discrete phylogeographic inference. Except for the intervention strategy preventing lineages spread to administrative areas with population sizes >1,000k, these results are consistent to those presented in Figures 1a-c and Supplementary Figures 1a-d albeit associated with higher credible intervals. The specific case of the intervention strategy preventing lineage spread to administrative areas of more than 1,000k people illustrates the limitation of the reduction in epidemic duration as a metric to investigate such scenarios. Indeed, this metric can be solely determined by the maintenance, after the pruning step, of a single lineage reaching the end of the real epidemic. As this is the case for most of the pruned trees (but not for the MCC tree) in that particular intervention strategy, no pronounced impact is detected on epidemic duration. Overall, this aspect illustrates that the epidemic size reduction is a more relevant and more robust metric than the epidemic duration reduction. Due to computational limitations, these alternative results and related credible interval are only based on 350 (instead of 1,000) posterior trees.

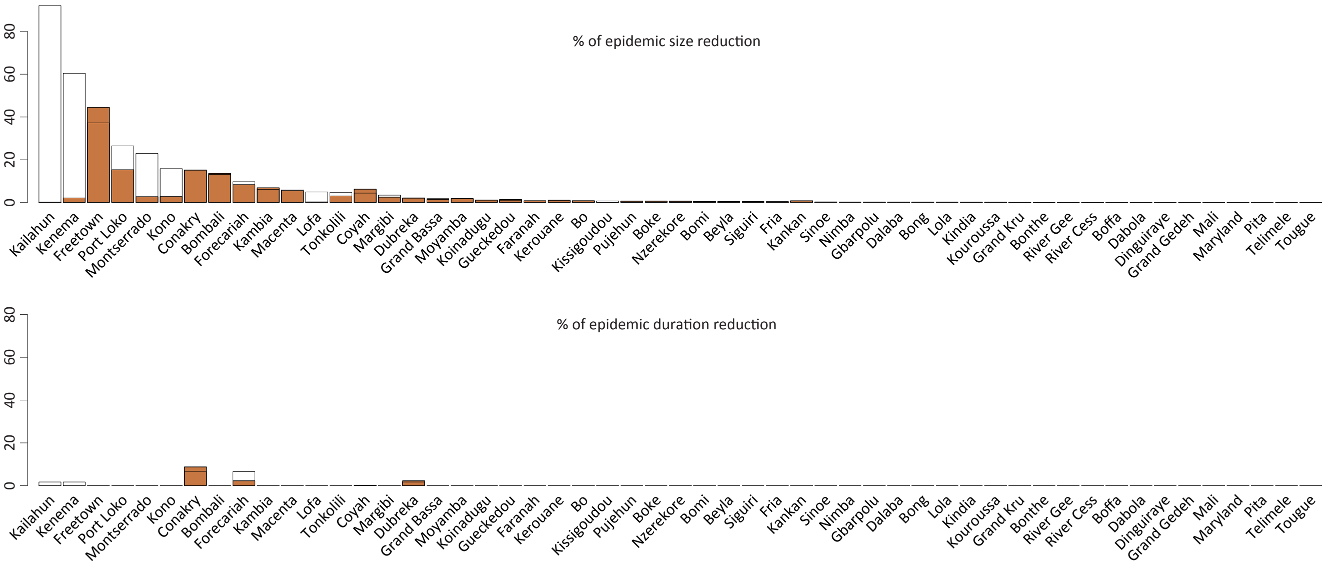


**Supplementary Figure 3. Epidemic size and duration reductions for different intervention scenarios in which introductions into a particular location have been prevented.** For each scenario, the effect on epidemic size and duration is quantified by comparing (in %) the tree length and height to the original phylogenetic tree. As in Figures 1c-d, we also report corresponding epidemic size reductions when transmission is prevented only after June 2014 (brown histogram).

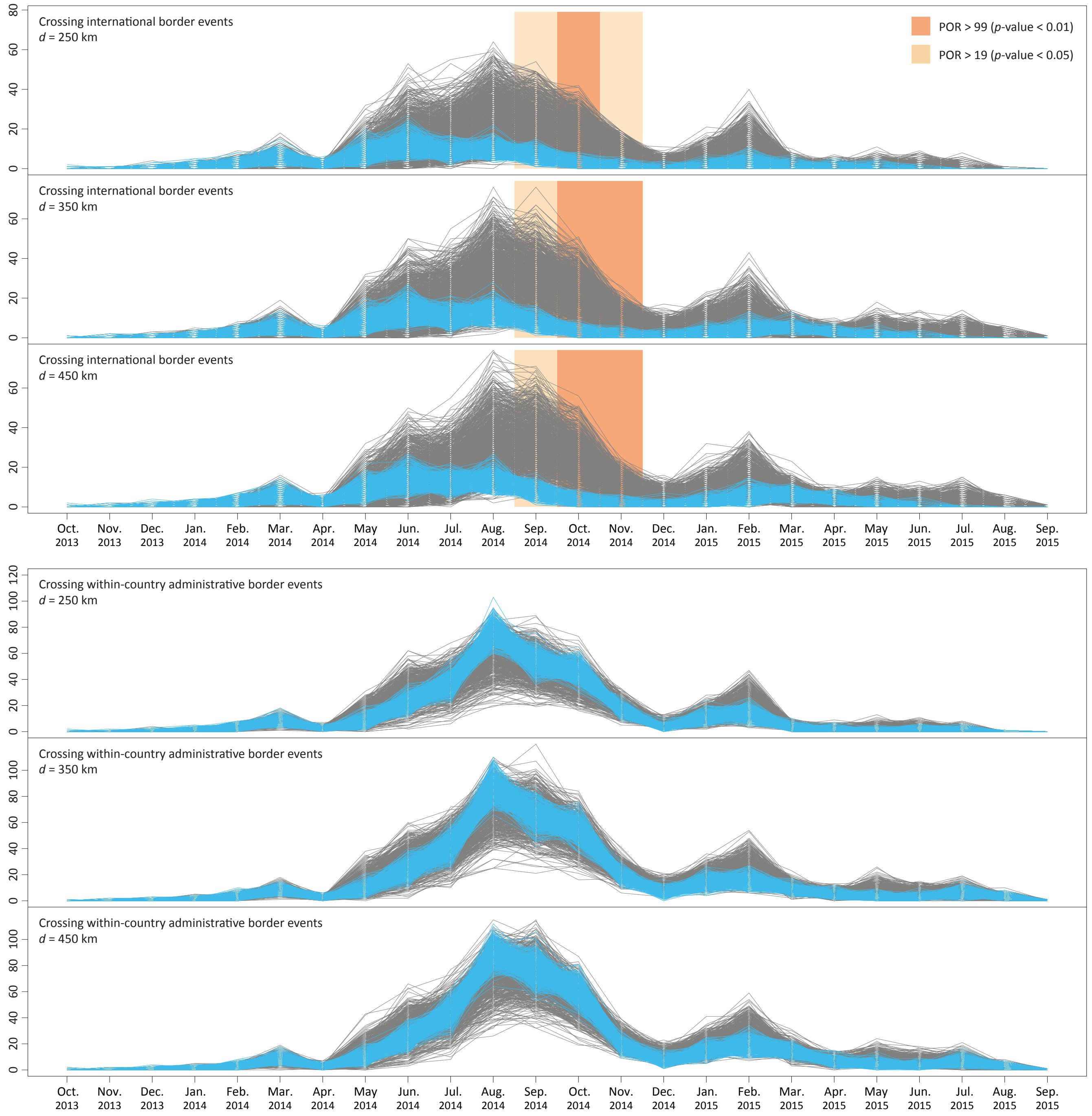


**Supplementary Figure 4. Detailed results for assessing the monthly impact of borders on the EBOV dispersal frequency.** These graphs display the monthly evolution of observed (in blue) and simulated (in grey) occurrences for each data set ($d$ = 250, 350 and 450 km). As described in the text, our posterior predictive simulation procedure is unaware of borders. As we do not expect any significant impact of within-country administrative borders on dispersion frequency, predictive odds ratio (POR) estimates for these borders are also included as a negative control. In addition, months for which the number of observed crossing border events is significantly smaller, i.e. associated with PORs $>$19 (or posterior predictive $p$-values $<$ 0.05), are highlighted in orange.

**Supplementary Note 1: estimating EBOV dispersal velocity based on a data set of 452 genomes from Sierra Leone with admin-3 level sampling precision.** In order to assess to what extent dispersal velocity estimates are sensitive to reducing clusters specific for an administrative area to a single representative sequence (with a randomly drawn sampling coordinate within that area), we performed an additional analysis based on an alternative data set of genome sequences from Sierra Leone. This data set constitutes all genomes drawn from patients with known disease outcome studied by Li *et al.*[1]. 447 out of 452 sequences in this data set have a known admin-3 level (chiefdom) of sampling. Given the relatively small size of these admin-3 polygons, each sequence was simply associated with the sampling coordinates of the centroid point of its polygon of origin (Supplementary Figure 5). For the five remaining sequences only associated with an admin-2 polygon of origin (district), their sampling coordinates were integrated out in the MCMC over a polygon that represents the relevant area[2]. We analysed this set of geo-referenced genomes with the continuous diffusion model implemented in BEAST[3]. As for the other continuous diffusion analyses performed in this study, we analysed the data set using a relaxed random walk (RRW) model with an underlying Cauchy distribution to represent among-branch heterogeneity in branch velocity (Lemey *et al.* 2010). We ran an MCMC chain using BEAST 1.8.4[4] for 100 million iterations, removing the first 5% of samples as burn-in. The output was analysed with Tracer 1.7 and revealed an estimated dispersal velocity of 2.12 km/day (95% HPD [1.95, 2.33]). This estimate is very similar to estimates obtained for the overall data set when only considering lineages sampled before December 2014, i.e. 1.90 km/day (95% HPD [1.75, 2.09]) for $d$ = 250km, 1.93 km/day (95% HPD [1.78, 2.11]) for $d$ = 350km, and 1.98 km/day (95% HPD [1.82, 2.12]) for $d$ = 450km.

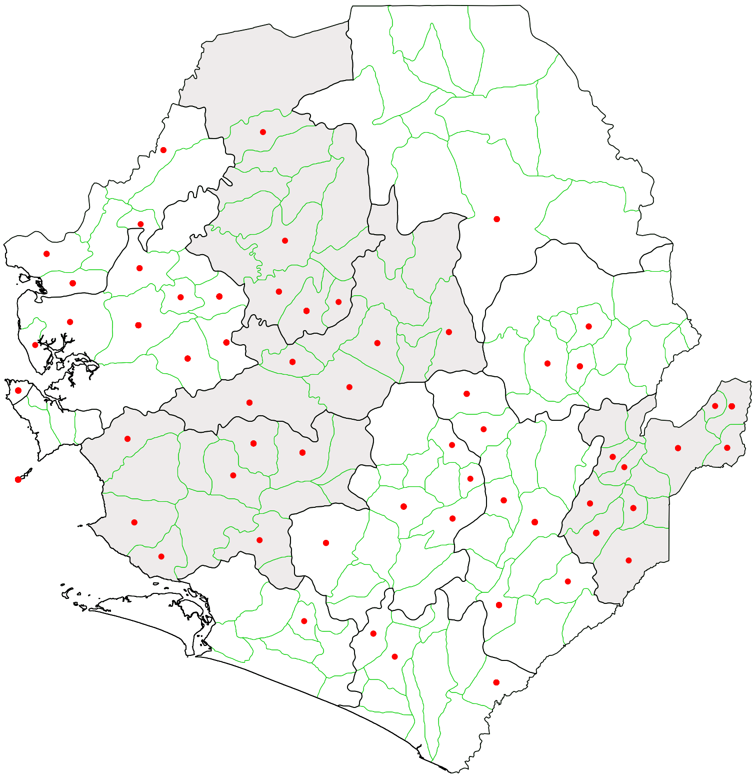

**Supplementary Figure 5. Sampling map of the alternative Sierra Leone data set.** Polygons with black and green contours represent the districts (admin-2) and the chiefdoms (admin-3) of Sierra Leone, respectively. Red dots correspond to centroid points of admin-3 polygons in which genome sequences have been sampled. For the analysis, geographic coordinates of these centroid points have been assigned to sequences originating from those admin-3 polygons. For five samples, only the admin-2 (instead of admin-3) polygon of origin was known. The four admin-2 polygons of origin for these EBOV genomes are shaded in grey (with two samples originating from the most northern grey polygon, i.e. Bombali District). For these four sequences, the corresponding admin-2 polygon has been specified as the area of origin and their sampling coordinates have been integrated over this polygon using MCMC (Nylinder *et al.* 2014).

## Supplementary References


1. Li, T. et al. Mapping the clinical outcomes and genetic evolution of Ebola virus in Sierra Leone. *JCI Insight* **2**, e88333 (2017).
2. Nylinder, S. et al. On the biogeography of Centipeda: A species-tree diffusion approach. *Syst. Biol.* **63**, 178-191 (2014).
3. Lemey, P., Rambaut, A., Welch, J. J., Suchard, M. A. Phylogeography takes a relaxed random walk in continuous space and time. *Mol. Biol. Evol.* **27**, 1877-1885 (2010).
4. Drummond A. J., Suchard M. A., Xie D., Rambaut, A. Bayesian phylogenetics with BEAUti and the BEAST 1.7. *Mol. Biol. Evol.* **29**, 1969-1973 (2012).
